# Utility of an In-Vitro Micro-Neutralizing Test in Comparison to a Plaque Reduction Neutralization Test for Dengue Virus, Japanese Encephalitis Virus, and Zika Virus Serology and Drug Screening

**DOI:** 10.3390/pathogens13010008

**Published:** 2023-12-20

**Authors:** Kazumi Haga, Zhenying (Nancy) Chen, Misao Himeno, Ryuichi Majima, Meng Ling Moi

**Affiliations:** 1 Department of Developmental Medical Sciences, Graduate School of Medicine, The University of Tokyo, Tokyo 113-0033, Japan; kazumihaga@m.u-tokyo.ac.jp (K.H.); mhimeno@m.u-tokyo.ac.jp (M.H.);; 2 Department of Biology, Emory College of Art and Science, Emory University, Atlanta, GA 30322, USA; nancy.chen2@emory.edu

**Keywords:** flavivirus, microneutralization test, neutralizing antibodies, dengue, Japanese encephalitis virus, Zika

## Abstract

Flavivirus infections, including dengue virus (DENV), Japanese encephalitis virus (JEV), and Zika virus (ZIKV), present significant global public health challenges. For successful vaccine design, the assessment of neutralizing antibody activity requires reliable and robust methodologies for determining antibody titers. Although the plaque reduction neutralization test (PRNT) is commonly acknowledged as the gold standard, it has limitations in terms of time and cost, and its usage may be limited in resource-limited settings. To address these challenges, we introduced the micro-neutralization test (MNT) as a simplified alternative to the PRNT. The MNT employs a 96-well plate format, conducts microscale neutralization assays, and assesses cell viability by dissolving cells to create a uniform color solution, which is measured with a spectrometer. In this study, we evaluated the utility of the MNT by contrasting the end-point titers of the MNT and PRNT using 4 monoclonal antibodies, 15 non-human primate serum samples, and 2 therapeutic drug candidates across flaviviruses. The results demonstrated a strong correlation between the MNT and PRNT titers, affirming the robustness and reproducibility of the MNT for evaluating control measures against flaviviruses. This research contributes valuable insights toward the development of a cost-effective antibody titer testing approach that is particularly suitable for resource-limited settings.

## 1. Introduction

Flaviviruses, which encompass a family of mosquito-borne viruses, such as dengue virus (DENV), Japanese encephalitis virus (JEV), and Zika virus (ZIKV), have posed global health challenges for the past decades [1,2,3,4]. These viruses are primarily transmitted by arthropods such as mosquitoes and ticks [5,6,7,8], resulting in the geographic distribution of these viruses being predominantly observed in tropical and subtropical areas [9,10]. Typically resulting in a pronounced fever, frequently exceeding 40 °C, infections caused by flaviviruses are challenging to diagnose during the acute phase due to their shared symptoms with other flavivirus infections. However, certain flaviviruses, like DENV, JEV, and ZIKV, may display distinctive clinical symptoms that aid in earlier diagnosis [11,12,13]. While the population at risk keeps increasing, no specific antiviral therapies currently exist, leaving only supportive care and symptomatic management as the mainstay of treatment [14]. With the pressing need for an efficacious anti-flavivirus vaccine, comprehensive understanding of neutralizing antibody activity assumes paramount importance. Consequently, the establishment of robust methodologies for determining neutralizing antibody titers is pivotal for vaccine development.

**Figure 1:**
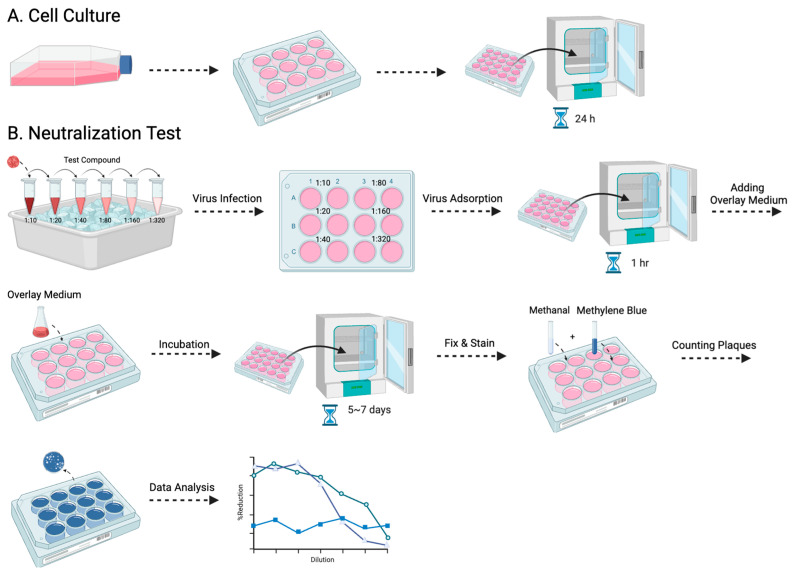
The procedure for the PRNT is presented in this flowchart: (**A**) entails the cultivation of Vero-9013 cells on a 12-well plate, incubated at 37 °C for 24 h in a pre-prepared medium, while (**B**) corresponds to the neutralization phase. The virus and serially diluted testing samples are pre-incubated on ice for 30 min. The sample virus mixtures are then introduced to the pre-cultured cells. After 1 h of virus adsorption, overlay medium is added and the plate is incubated for 5 to 7 days. The cells then undergo fixation with methanol, staining with methylene blue, and the formed plaques are counted by the naked eye.

**Figure 2:**
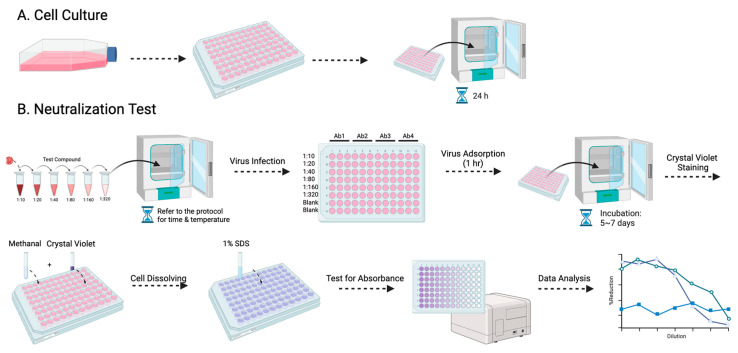
The procedure for the MNT is presented in this flowchart: (**A**) entails the cultivation of Vero-9013 cells on a 96-well plate, incubated at 37 °C for 24 h in a pre-prepared medium, while (**B**) corresponds to the neutralization phase. The virus and serially diluted testing samples are pre-incubated under specified conditions (refer to the protocol for details). The sample virus mixtures are then introduced to the pre-cultured cells. Following 1 h of virus adsorption and 5–7 days of incubation, the cells undergo fixation with methanol, staining with crystal violet, dissolution with 1% SDS, and measurement of absorbance at 595 nm using a spectrometer.

Among the various antibody titer tests, the plaque reduction neutralization test (PRNT) is a widely used gold standard method, primarily employed to accurately quantify the number of non-neutralized viruses present at a specific antibody concentration [15]. However, despite being a valuable and widely utilized tool, the PRNT does present certain limitations, as other studies have highlighted as well [16,17]. The complex process typically takes approximately a week to complete, and the associated expenses involved can be substantial (Figure 1). Furthermore, to visualize the plaques, a 12-well plate would be needed to perform the PRNT, which limits the efficacy of testing the neutralizing level on a larger scale. Here, we evaluated a simplified approach using the micro-neutralization test (MNT) to determine the required antibody titer for virus neutralization, which has higher efficacy, easier procedures, and better accuracy. By staining the infected cell culture and subsequently dissolving the cells, we can exploit the differential dye uptake between living cells and dead infected cells to accurately measure the concentration of the cell solution (Figure 2).

Our study aims to conduct a comparative analysis between the MNT and the PRNT for evaluating antibody titers against DENV, JEV, and ZIKV. Within this paper, we provide a detailed description of both testing methods when employed using the Vero-9013 cell line, 4 different types of monoclonal antibodies, 15 marmoset serum samples, and 2 therapeutic drug candidates. The outcomes of this study contribute valuable insights toward the development of a simpler and more cost-effective approach for antibody titer testing, with potential applicability and relevance to flavivirus-affected regions in a limited-resource setting.

## 2. Materials and Methods

### 2.1. Cells and Viruses

Vero-9013 cells derived from African green monkey kidneys were cultured in Eagle’s minimum essential medium (EMEM) supplemented with 10% heat-inactivated fetal bovine serum (FBS) without antibiotics. The cells were maintained in a 37 °C incubator with 5% CO_2_.

The study employed dengue virus type-1 (DENV-1) 01-44-1HuNIID strain (GenBank accession no. AB111070), Japanese encephalitis virus (JEV) OH0566 strain (GenBank accession no. AY508813), and Zika virus (ZIKV) PRVABC59 strain (GenBank accession no. KX377337). These viruses were cultured on Vero-9013 cells for 5 days at 37 °C with 5% CO_2_. Afterwards, the cell culture supernatant was harvested, clarified through centrifugation, and stored in several aliquots at −80 °C. The virus titers were measured in plaque-forming units (PFU) per mL by performing plaque assays on the Vero 9013 cells.

### 2.2. Monoclonal Antibodies, Serum Samples, and Therapeutic Drug Candidates

Both neutralization assays utilized multiple types of mouse monoclonal IgG antibodies (200 μg/mL). The monoclonal antibodies used included 4G2 (MAbs, ATCC MAb HB-112 D1-4G2-4-15), demonstrating reactivity across flaviviruses. Additionally, the monoclonal antibodies B247, 12D11, and B376 were produced in-house and were specifically designed for neutralizing DENV. Serum samples were collected from marmosets that had been previously infected with live-attenuated DENV-1 16007 PDK-13 and DENV-2 16681 PDK-5. Bred for research purposes, the common marmosets (*Callithrix jacchus*) utilized in this study were donated by CLEA Japan, Inc., Tokyo, Japan All the animal-related procedures adhered to the guidelines of the Institutional Animal Care and Use Committee of NIID, Tokyo, Japan. Ribavirin and glycyrrhizin (Wako Pure Chemical Corporation, Tokyo, Japan) was dissolved in DMSO at a concentration of 500 mg/mL and stored at −20 °C.

### 2.3. Plaque Reduction Neutralization Test

Following serial 2-fold dilutions, the monoclonal antibodies, marmoset serum, or therapeutic drug candidates were combined with the virus at a 1:1 ratio and incubated at 4 °C for 30 min, according to a previously described method [18]. The resulting mixture was then introduced into the Vero-9013 cells in 12-well plates. After a 1 h adsorption period, 1.5 mL of overlay medium (1% methylcellulose with EMEM 2% FBS) was added to each well. The plates were left to incubate in 5% CO_2_ at 37 °C until the plaques became clear and countable. The cells were fixed with 4% paraformaldehyde in phosphate-buffered solution (Wako Pure Chemical Corporation, Tokyo, Japan) for 1 h at room temperature and subsequently stained with 0.375% methylene blue (Wako Pure Chemical Industries). Plaque counting was performed visually, and the neutralizing titer was determined as the inverse serum dilution where the plaque counts were below 50% of the designated cut-off (≥50% inhibition).

### 2.4. Micro-Neutralization Test

Following serial 2-fold dilutions, the monoclonal antibodies, marmoset serum, or therapeutic drug candidates were combined with the virus at a 1:1 ratio and incubated at 4 °C for 30 min or 37 °C for 1 h in the case of therapeutic drug candidates. The resulting mixture was then introduced into the Vero-9013 cells in 96-well plates. After a 1 h adsorption, the plates were incubated in 5% CO_2_ at 37 °C for 5–7 days. The cells were fixed with methyl alcohol (Wako Pure Chemical Industries) for 10 min at room temperature and subsequently stained for 15 min with 0.1% crystal violet (Wako Pure Chemical Industries). Excess dye was washed, and 1% sodium dodecyl sulfate (SDS) was applied to dissolve the cells. Absorbance was tested using 595 nm light. The neutralizing titer was determined as the inverse serum dilution where the absorbance of 595 nm light was below 50% of the designated cut-off (≥50% inhibition).

### 2.5. Data Analysis

The MNT calculations were performed separately for each plate. The virus control wells were required to attain a mean OD_595 nm_ (Optical Density under the light of 595 nm) within the range of 1.0 to 3.0, while the cell control was expected to have a median OD_595 nm_ of approximately 2.0. The following equation was utilized to determine the percent reduction in dead cells for each plate:

(1) $$\mathrm{Percent}\;\mathrm{Reduction}=\frac{\left(1-\frac{{mean\;OD}_{595\;nm}\;blank}{{mean\;OD}_{595nm}\;control}\right)-\left(1-\frac{{mean\;OD}_{595nm}\;experimental}{{mean\;OD}_{595nm}\;control}\right)}{1-\frac{{mean\;OD}_{595nm}\;negative\;control}{{mean\;OD}_{595nm}\;control}}\;=\frac{mean\;{OD}_{595nm}\;experimental-{mean\;OD}_{595nm}\;blank}{{mean\;OD}_{595nm}\;control-{mean\;OD}_{595nm}\;blank}$$

In the calculation of the percent reduction in dead cells using the MNT, three essential groups are considered: blank (cells only), control (cells and virus), and experimental (cells, virus, and antibody). Normalizing the OD_595 nm_ of the control with the negative control enables the determination of the percentage of viable cells in the presence of the virus. The ratio of the OD_595 nm_ of the experimental group to the control group represents the percent of viable cells due to the neutralization effect. Subtracting this ratio from 100% yields the percentage of dead cells. Consequently, the percent reduction in dead cells can be defined as the difference in the percentage of dead cells between the blank group and the experimental group, as normalized by the percentage of dead cells in the blank group.

To account for drug-induced cell cytotoxicity, the calculation of the percentage reduction in cell death varies slightly from the previous equation when assessing the effect of antibodies and serum on viruses. Note that when determining the percentage of viable cells in the experimental group, the mean OD_595 nm_ of the experimental group is divided by the mean OD_595 nm_ of a control group with an equivalent drug concentration. Consequently, the updated calculation equation for drugs is as follows:

(2) $$\mathrm{Percent}\;\mathrm{Reduction}=\frac{\left(1-\frac{{mean\;OD}_{595nm}\;blank}{{mean\;OD}_{595nm}\;control}\right)-\left(1-\frac{{mean\;OD}_{595nm}\;experimental}{{mean\;OD}_{595nm}\;control\;with\;drug}\right)}{1-\frac{{mean\;OD}_{595nm}\;control}{{mean\;OD}_{595nm}\;blank}}$$

Statistical analyses were performed using R version 4.2.2. The data underwent logarithmic transformations to achieve a normal distribution of neutralizing titers among the trials. The normal distribution was re-ensured via a quantile-quantile plot, and the correlation between the MNT and PRNT was evaluated using the Pearson correlation test.

## 3. Results

### 3.1. Evaluation of the Microneutralization Test

**Figure 3:**
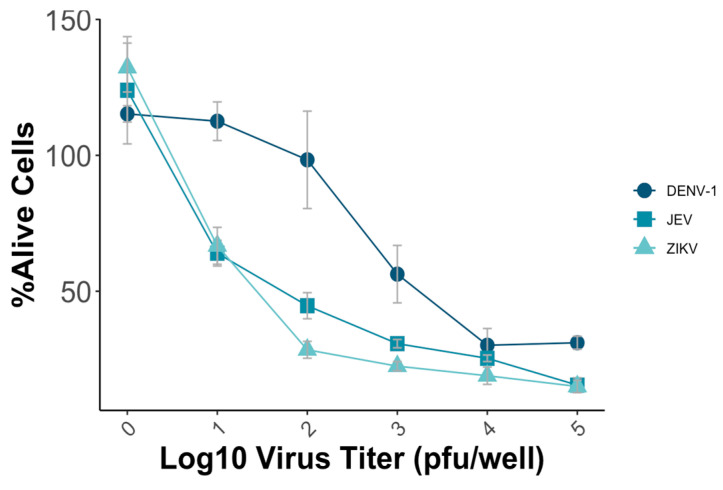
Vero-9013 cells were exposed to varying virus titers of DENV-1, JEV, and ZIKV, ranging from 10 pfu/well to 10^5^ pfu/well using a 10-fold dilution series. OD_595 nm_ measurements were performed for the experimental group and control group, from which the percentage of viable cells was subsequently calculated. Each data point represents the geometric mean derived from three replicate measurements, with the error bars denoting the standard deviation among these replicates.

To establish the MNT, various parameters, including the virus titer and incubation time, were systematically evaluated to optimize the test’s sensitivity, reproducibility, and efficiency. Vero-9013 cells were infected with different predetermined virus titers using a 96-well plate, ranging from 10 pfu/well to 10^5^ pfu/well with a 10-fold dilution. The optimal condition for conducting the MNT involved achieving a mean percentage of alive cells of around 50%, which ensures the subsequent detection of the neutralizing effect of the viruses. The measured OD in each well represented the viability of Vero-9013 cell cultures exposed to mouse monoclonal antibodies that were serially diluted. The percentage of viable cells was calculated by comparing the OD_595 nm_ value of the experimental group with the OD_595 nm_ value of the control group on the same plate. In this study, the plates that used JEV and ZIKV were incubated for 4 days, while those with DENV-1 were incubated for 5 days. For DENV-1, a virus titer of 5 × 10^3^ pfu/well resulted in approximately 50% viability, while for JEV and ZIKV, titers of 10^2^ pfu/well and 5 × 10 pfu/well, respectively, achieved a similar percentage (Figure 3).

### 3.2. Determination of the MNT Titers Using Monoclonal Antibodies

Following the optimization of the virus incubation conditions, the MNT was conducted using mouse flavivirus cross-reactive monoclonal antibodies. The percentage reduction in cell death was calculated after the application of the monoclonal antibodies 12D11, 4G2, B247, and B376 to determine their neutralizing effects against DENV-1, JEV, and ZIKV (Figure 3). Consistent with the results obtained from the PRNT, the monoclonal antibodies 12D11, 4G2, and B376 exhibited higher neutralizing activity against DENV-1, while the monoclonal antibody B247 demonstrated limited neutralization activity across all the tested viruses (Table 1). The monoclonal antibodies used in this study have higher neutralizing activity for DENV but lower activity against JEV and ZIKV, which is relatively weak. Nevertheless, both the MNT and PRNT consistently demonstrated their robustness in evaluating the neutralizing effects of the monoclonal antibodies.

### 3.3. Determination of the MNT Titers Using Serum Samples

To evaluate the utility of the MNT, neutralization tests were performed with 15 serum samples from marmosets that had been previously infected by DENV. All the samples demonstrated a strong neutralizing effect in DENV-1 for both the MNT and the PRNT (Table 2).

When using the MNT, the observed antibody titers often appear higher than those from the PRNT due to methodological differences. Unlike the PRNT, which employs an overlay medium to restrict virus spread, the MNT allows virus infection in all the well cells, leading to more plaque and reduced cell concentration. Consequently, the recorded absorbance values are lower, resulting in higher calculated antibody titers. In essence, the MNT emulates a scenario that closely resembles in vivo conditions, theoretically enhancing the accuracy of the antibody titer measurements.

### 3.4. Determination of the MNT Titers Using Antiviral Drug Candidates

In instances where no specific cure exists for viruses like flaviviruses, alternative antiviral therapies are frequently employed [19,20,21,22]. Both ribavirin and glycyrrhizin are potential antiviral drugs showing promise against flaviviruses according to previous studies [23]. In this context, we evaluated the impact of both drugs on DENV-1, JEV, and ZIKV using the MNT.

**Figure 4:**
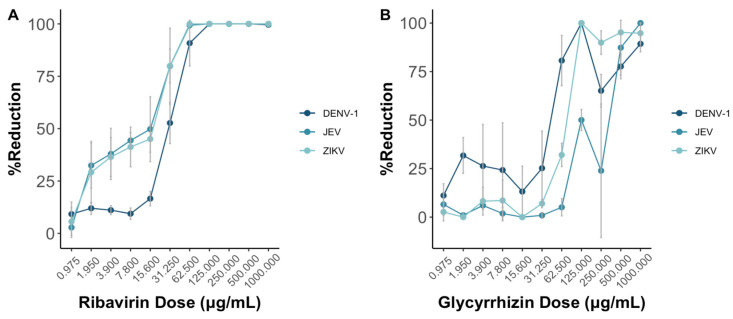
Ribavirin (**A**) and glycyrrhizin (**B**) were used to test the antiviral effects against DENV-1, JEV, and ZIKV through the MNT using Vero-9013 cells. Both ribavirin and glycyrrhizin were diluted in 2-folds ranging from 1000 μg/mL to 0.975 μg/mL. The percent reduction is calculated by the OD_595 nm_ values of the experimental group, positive control group, negative group, and control group as shown in Equation (2). Each data point represents the geometric mean derived from six replicate measurements, with the error bars denoting the 95% confidence intervals among these replicates.

As the doses of both ribavirin and glycyrrhizin increase, the presented reduction in virus death shows a progressive rise in the percentage reduction of cell death across DENV-1, JEV, and ZIKV (Figure 4). Notably, this effect appears more pronounced for both JEV and ZIKV in comparison to DENV-1 when ribavirin was used.

### 3.5. Comparison of the MNT to the PRNT

Both the MNT and PRNT were employed to test for the neutralizing titers for four monoclonal antibodies and two drugs against DENV-1, JEV, and ZIKV. Additionally, the neutralizing antibody titers for DENV-1 in 15 serum samples were determined using both methods as well. Across all the virus types, ribavirin and glycyrrhizin displayed antiviral efficacy exceeding 50% at a concentration of 31.25 μg/mL and 125 μg/mL, respectively, using the MNT. Similarly, using the PRNT, the half maximal effective concentrations (EC_50_) of ribavirin and glycyrrhizin were 500 μg/mL and 250 μg/mL, respectively. In this context, further studies are required to validate the EC_50_ as determined via plaque assay in comparison to the MNT and other tests that include cell viability as the total activity.

**Figure 5:**
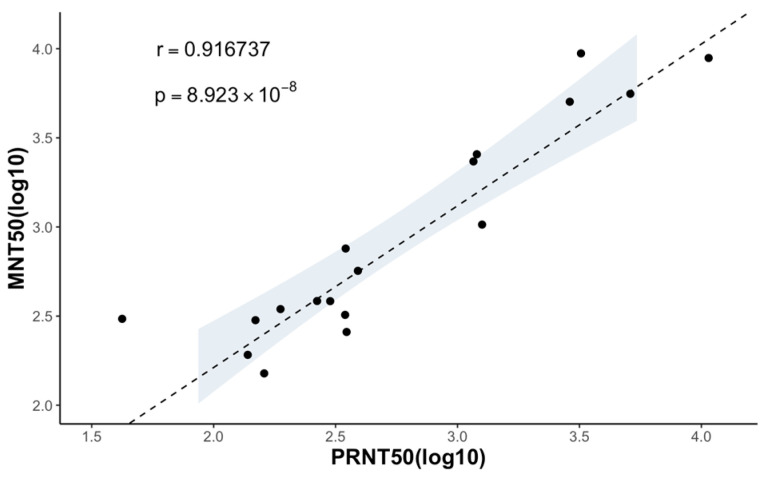
Correlation between the neutralizing titers measured using the MNT and PRNT. MNT_50_ and PRNT_50_ of 4 anti-flavivirus mouse monoclonal antibodies and 15 serum samples are plotted in a logistic scale, and the correlation between the two is calculated (r=0.916737), with a *p* value significantly smaller than 0.05. The 95% confidence interval of the linear regression is shown in the shaded area.

Since the coefficient of determination (r) of 0.9167 between the MNT_50_ and the PRNT_50_ for the monoclonal antibodies and marmoset serum samples was obtained, we concluded that there is a strong correlation between the results of the MNT and PRNT (Figure 5).

## 4. Discussion

Mosquito-borne flaviviruses pose a significant global public health threat for which there is currently no specific cure [1,14]. Therefore, the development of a vaccine is of paramount importance and urgency. As neutralizing antibodies are pivotal in either preventing flavivirus infection or mitigating the disease severity, it becomes crucial to assess their impact when evaluating the effectiveness of potential flavivirus vaccines [24,25,26,27]. In this research, the proposed MNT accurately determined the neutralizing capacity of the monoclonal antibodies and serum samples, as well as the effectivity of the therapeutic drugs. The MNT delivered robust and consistent outcomes, aligning with the PRNT, which is acknowledged as the gold standard for determining neutralization titers [15]. Moreover, the MNT exhibited versatility by accommodating multiple viruses, including DENV, JEV, and ZIKV. This versatility underscores the method’s suitability for gauging neutralization titers in the context of mosquito-borne flaviviruses.

The MNT quantifies the percentage of viable cells that have survived under conditions where a portion of the viruses has been neutralized or rendered inactive. This percentage of viable cells is then converted into the corresponding percentage of deceased cells, simplifying the analysis and interpretation of the MNT results. In essence, the cells were cultured with non-neutralized or active viruses in a 96-well plate, followed by fixation and dissolution. The MNT operates on the principle that optical changes can detect increases in cell viability and reductions in cell death resulting from antibody neutralization or the inactivation of antiviral drugs [28,29]. Within this framework, the approach to analyzing the percentage of cell death caused by non-neutralized or active viruses offers valuable insights into the infection rates of these viruses.

In assessing the effectiveness of the MNT in determining the neutralizing titers for representative mosquito-borne flaviviruses, we employed mouse monoclonal antibodies targeting flavivirus and marmoset serum samples. The MNT end-point titers (MNT_50_) exhibit a strong correlation with the PRNT end-point titers (PRNT_50_), as evidenced by a coefficient of determination (*r*) of 0.9167 (Figure 5). The findings from the MNT indicate that it offers a technically straightforward alternative to the PRNT, with results that are in line with those obtained using the PRNT. It is worth noting that the MNT typically yields higher neutralization titers compared to the PRNT (Table 2). This might be because the MNT allows the virus to infect all the cells, generating more plaques and a lower cell concentration. The resulting calculated antibody titers are thus higher, closely mimicking in vivo conditions and potentially enhancing accuracy.

Beyond assessing neutralization titers, the MNT also serves as a tool for determining the effective concentration of antiviral drugs, such as ribavirin and glycyrrhizin, required to mitigate virus-induced cytopathogenicity. Given that drugs can induce cell cytotoxicity, the assessment of the dose effect traditionally involves two parameters: EC_50_, indicating the concentration necessary to reduce virus-induced cytopathogenicity by 50%, and CC_50_, representing the 50% cytotoxic concentration [30]. However, due to the nature of the MNT, where cell viability is directly measured, the influence of cytotoxicity can be eliminated, and the direct calculation of the drug-induced reduction in infectivity becomes possible. With the experimental group data and cytotoxicity data involving various drug concentrations applied directly to the cells, the impact of cytotoxicity is nullified as each experimental group corresponds to a specific control group with an assigned drug amount.

The potential breadth of the MNT’s applicability extends beyond that of the PRNT, primarily due to its independence from the necessity of distinct plaque formation for accurate counting. First, the MNT can be employed in a broader range of virus and cell line combinations compared to the PRNT, given that the MNT does not necessitate distinct plaque formation for counting. The variation in plaque morphology formed using the PRNT, depending on the virus and cell line, can lead to difficulties in counting and introduce human biases. For example, DENV-3 and DENV-4 in Vero-9013 cells form particularly small plaques, while JEV plaques are often blurry regardless of the cell line used. The MNT addresses this by not requiring plaque counting, enabling antibody titers to be detected using a spectrometer. Second, in evaluating antiviral drug efficacy, the PRNT faces the challenges of unclear plaques, particularly when using clinical samples. The MNT, in contrast, bypasses the need for plaque counting, is less laborious and allows for rapid quantification.

The MNT offers several potential advantages over the traditional PRNT, including enhanced efficiency, reduced costs, and improved accuracy. Unlike the PRNT, which requires cell plates to be incubated in a 12-well format for adequate plaque growth and subsequent counting, the MNT allows for the use of a 96-well plate, enabling rapid assessment of neutralizing titers for a larger number of serum samples. Furthermore, while the overall expenses for one plate of the PRNT and MNT are approximately equal, the MNT’s efficiency is 8 times higher, as it can be performed in a 96-well plate rather than a 12-well plate—making the cost of collecting data from 1 well around 8 times lower than the PRNT. Regarding time, the MNT eliminates the 1 h virus adsorption and plaque counting steps, which typically take 10 to 20 min per plate on average in the PRNT. Therefore, the MNT substantially reduces the time required for experiments due to its simplified procedure, especially when dealing with a large quantity of samples. Additionally, our tests indicate that, under conditions of relatively high initial virus titer and antibody concentration during pre-incubation, the MNT only necessitates a 3-day virus adsorption period compared to the conventional PRNT, which requires 5 days. Moreover, the PRNT lacks standardization and uniformity across various laboratories [31], leading to potential biases stemming from the labor-intensive manual plaque counting process. In comparison to other micro-neutralization tests that targeting other viruses such as SARS-CoV-2, we find our results and conclusions aligning with theirs, further proving the effectiveness of the MNT across various viruses. Moreover, studies of other MNTs also emphasized the enhanced throughput and reduced operator workload enabled by MNT-like assays, reinforcing the utility of this approach [32,33,34]. Collectively, these advantages position the MNT as a viable alternative method, particularly suitable for laboratories with limited resources where flavivirus is most prevalent, to conduct serology and drug screening assays.

## 5. Conclusions

A new testing approach, the MNT, was developed to assess the neutralizing antibody titers of key mosquito-borne flaviviruses, encompassing DENV, JEV, and ZIKV. The efficacy of the MNT was assessed using 4 distinct monoclonal antibodies, 15 marmoset serum samples, and 2 antiviral drug candidates. A strong correlation between the MNT and the PRNT results underscores the robustness and replicability of this innovative test, presenting a feasible high-throughput alternative to the gold standard PRNT. Because of the MNT’s distinctive approach of directly measuring cell viability, it can mitigate the impact of cytotoxicity and enable the direct calculation of drug-induced reductions in infectivity. Given its compatibility with a 96-well plate format and simplified procedures, the MNT substantially reduces costs while markedly enhancing time efficiency. Also, the simplicity of the MNT, combined with its reliance on machine measurements, minimizes the need for extensive training and enhances results’ accuracy. The MNT holds the potential to serve as a versatile tool for fundamental research and diagnostic applications, expediting the pace of neutralization assay throughput in both epidemiological investigations and vaccine studies. The MNT is particularly impactful in strengthening diagnostic capabilities and the routine monitoring of flavivirus infections, particularly in countries with limited resources.

## Figures and Tables

**Table 1:** Neutralizing titers of monoclonal antibodies (MNT_50_ vs. PRNT_50_) to DENV, JEV, and ZIKV.

Virus	mAb 12D11		mAb 4G2		mAb B247		mAb B376	
	MNT_50_ ^1^	PRNT_50_ ^2^	MNT_50_	PRNT_50_	MNT_50_	PRNT_50_	MNT_50_	PRNT_50_
DENV-1	640	1280	160	160	<10 ^3^	<10	1280	2560
JEV	<10	<10	<10	<10	<10	<10	<10	<10
ZIKV	<10	<10	<10	<10	<10	<10	<10	<10

^1^ MNT_50_ end-points were calculated by taking the inverse of the highest antibody dilution that reduced the corresponding OD_595 nm_ by at least 50%, comparing it to the control wells that have no antibodies applied; ^2^ PRNT_50_ end-points were calculated by taking the inverse of the highest antibody dilution showing at least 50% reduction in the corresponding plaque counts in experimental wells, comparing it to the control wells that have no antibodies applied; ^3^ Neutralizing titers that are less than 10 represent the case where the titers are below the detection limit of the assay. DENV-1 samples were serially diluted two-fold at a time, from 1:10 to 1:10,240, while the JEV and ZIKV samples were serially diluted two-fold at a time, from 1:10 to 1:640.

**Table 2:** Neutralizing titers of serum samples (MNT_50_ vs. PRNT_50_) to DENV-1.

Sample Code	DENV-1	
	MNT_50_ ^1^	PRNT_50_ ^2^
1	10,240	2560
2	5260	2560
3	10,240	10,240
4	2560	1280
5	320	160
6	1280	1280
7	5260	5260
8	320	320
9	320	40
10	320	320
11	640	320
12	640	320
13	320	320
14	320	320
15	320	160

^1^ MNT_50_ end-points were calculated by taking the inverse of the highest antibody dilution that reduced the corresponding OD_595 nm_ by at least 50%, comparing it to the control wells that have no antibodies applied; ^2^ PRNT_50_ end-points were calculated by taking the inverse of the highest antibody dilution showing at least 50% reduction in the corresponding plaque counts in experimental wells, comparing it to the control wells that have no antibodies applied.

## Data Availability

The dataset is available upon reasonable request to the corresponding author.
